# Impact of hysteroscopy on pregnancy outcomes in women with endometriosis-associated infertility undergoing their first frozen-thawed embryo transfer: a retrospective cohort study

**DOI:** 10.3389/fmed.2026.1849361

**Published:** 2026-09-24

**Authors:** Yujie He, Xin Zhang, Yuheng Li, Qingyang Chen, Yichi Zhang, Zhijun Jin, Ningxia Sun

**Affiliations:** 1 Center for Reproductive Medicine, Shanghai Changzheng Hospital, Shanghai, China; 2 Obstetrics and Gynecology, Shanghai Changzheng Hospital, Shanghai, China

**Keywords:** endometriosis, frozen embryo transfer, hysteroscopy, pregnancy outcomes, uterine cavity lesions

## Abstract

**Background:**

Endometriosis is associated with infertility, adverse assisted reproductive outcomes, and a high prevalence of intrauterine pathologies. Lesions such as endometrial polyps and chronic endometritis may impair endometrial receptivity and embryo implantation. Although hysteroscopy is the gold standard for diagnosing and treating intrauterine lesions, its value before the first frozen embryo transfer (FET) in women with endometriosis-related infertility remains uncertain. This study evaluated whether hysteroscopic surgery before the first FET improved pregnancy outcomes.

**Methods:**

This single-center retrospective cohort study consecutively enrolled 220 infertile women with endometriosis undergoing their first FET at the Reproductive Medicine Center of the Shanghai Changzheng Hospital between January 1, 2021, and May 31, 2025. Patients were divided into hysteroscopy (*n* = 180) and non-hysteroscopy (*n* = 40) groups according to whether they underwent hysteroscopic evaluation and treatment before FET. Baseline characteristics and pregnancy outcomes were compared. To reduce imbalance, 1:1 propensity score matching (PSM) was performed, primarily based on embryo attributes, yielding 40 patients per group. Primary outcomes were clinical pregnancy and live birth rates.

**Results:**

Both before and after PSM, the hysteroscopy group exhibited higher clinical pregnancy rates than the non-hysteroscopy group [pre-PSM: 65.56% vs. 42.50%, *P* < 0.05; post-PSM: 67.50% vs. 42.50%, *P* < 0.05]. The live birth rate was also higher in the hysteroscopy group both before and after PSM [pre-PSM: 55.56% vs. 35.00%, *P* < 0.05; post-PSM: 55.00% vs. 35.00%, *P* > 0.05], although the post-PSM difference did not reach statistical significance. After PSM, the hysteroscopy group also showed a significantly higher biochemical pregnancy rate than the non-hysteroscopy group [75.00% vs. 52.50%, *P* < 0.05]. No significant differences were found in miscarriage rate, preterm birth rate, or multiple pregnancy rate between the two groups after matching. Hysteroscopic evaluation revealed a high incidence of intrauterine abnormalities among patients with endometriosis, with lesion rates of 82.78% before PSM and 87.50% after PSM.

**Conclusion:**

Intrauterine lesions, particularly endometrial polyps and chronic endometritis, are highly prevalent in endometriosis-related infertility. Hysteroscopic surgery before the first FET was associated with a higher clinical pregnancy rate. Given the high lesion prevalence, hysteroscopic evaluation before the first FET may be considered in these patients.

**Trial registration:**

This is a retrospective cohort study.

## Introduction

Endometriosis is characterized by the growth of active endometrial tissue, including glands and stroma, outside the uterus. Common symptoms include dysmenorrhea, chronic pelvic pain, and infertility (1). Studies have indicated that infertility occurs in up to 20% of women with laparoscopically confirmed endometriosis (2). This condition can lead to infertility by altering pelvic anatomy, impairing ovarian function, causing ovulation disorders, and reducing endometrial receptivity, all of which impact female conception (3). While assisted reproductive technology (ART) is an effective treatment for infertility related to endometriosis (4), the success rates of ART in these patients remain a clinical concern. Studies have shown that patients with endometriosis have significantly lower fertilization, implantation, and pregnancy rates compared with those without the condition (5–7). A retrospective study comparing IVF treatment for endometriosis versus tubal factor infertility included 433 patients (225 FET cycles) and 1,299 patients (697 FET cycles), respectively (8). The clinical pregnancy rate and live birth rate in the endometriosis group were lower than those in the tubal factor group (47.56% vs. 55.95%, and 33.78% vs. 44.47%, respectively), and the cumulative live birth rate was also significantly reduced in the endometriosis group (52.19% vs. 60.05%) (8). Despite the advancements in ART, a recent study that analyzed the impact of different endometriosis stages on cumulative pregnancy rates in IVF found that patients with severe endometriosis had significantly lower cumulative clinical pregnancy and sustained pregnancy rates compared with those without endometriosis and those with mild endometriosis (9).

Embryo implantation is a complex process involving interactions between the embryo and the endometrium, specifically focusing on embryo quality, endometrial receptivity, and the synchronization of embryo-endometrial development (10). Failures in embryo transfer may be linked to abnormalities in the uterine cavity, such as chronic endometritis (CE), endometrial polyps (EP), intrauterine adhesions, and uterine leiomyomas (11), as well as diminished endometrial receptivity (10). Endometriosis not only adversely affects oocyte quality (12) and reduces embryo quality but also potentially impacts endometrial receptivity, which can lead to unsuccessful embryo transfers (13). Endometrial receptivity refers to the ability of the endometrial tissue to support embryo implantation and initiate pregnancy (14). Research indicates that both organic and inflammatory lesions in the uterine cavity can compromise endometrial receptivity (15). Specifically, endometrial polyps and chronic endometritis can modify the microenvironment of the uterine cavity, negatively affecting endometrial receptivity and consequently hindering embryo implantation and normal development, which adversely impacts the outcomes of assisted reproduction (16–18).

It is worth noting that an increasing number of studies have indicated that the prevalence of EP and CE is higher in patients with endometriosis compared with those without (19–22). EP are benign hyperplastic lesions characterized by the excessive growth of local endometrial glands and stroma, which become re-epithelialized and protrude from the surrounding endometrium. These polyps can impair endometrial receptivity by mechanically interfering with the endometrium and releasing molecules that negatively affect sperm transport and embryo implantation, thereby adversely impacting fertility (23). Zhang et al. reported that the incidence of EP in patients with endometriosis was 47.83%, significantly higher than the 29.82% observed in the control group (19). CE is a mild, persistent inflammatory condition affecting the endometrium (24). Its classic features include micropolyps, interstitial edema, and focal or diffuse congestion, characterized by the infiltration of Cluster of Differentiation 138 (CD138)-positive endometrial stromal plasma cells (25, 26). A meta-analysis revealed that the prevalence of chronic endometritis in women with endometriosis was 28%, with a notably higher prevalence in patients with revised American Society for Reproductive Medicine (rASRM) stage III-IV (43%) compared with those with rASRM stage I-II (25%) (27). The meta-analysis also demonstrated that women with endometriosis had a significantly higher prevalence of chronic endometritis than controls (five studies, 264 endometriosis cases, 435 controls; OR = 2.07; 95% CI 1.11–3.84, I^2^= 43%, *P* = 0.02) (27). Takebayashi et al. found that the prevalence of CE, diagnosed through local immunostaining of CD138 in the endometrial stroma, was significantly higher in women with endometriosis compared with those without (52.94% vs. 27.02%) (20). Consequently, it is crucial for patients with endometriosis to rule out endometrial lesions such as EP and CE before undergoing frozen-thawed embryo transfer.

Abnormalities in the uterine cavity can significantly contribute to reduced fertility and failed implantation rates, as these lesions may hinder embryo implantation, thereby decreasing the clinical pregnancy rate. Ensuring a morphologically normal uterine cavity is essential for improving live birth and clinical pregnancy rates while minimizing early pregnancy loss (28–30). Hysteroscopy is the only technique that permits direct observation and simultaneous treatment of endometrial lesions (31). It serves both diagnostic and therapeutic roles for lesions affecting the anatomical structure of the uterine cavity and the endometrium. A recent meta-analysis by Vitale et al., which included 15 studies involving 5,038 women, found moderate-quality evidence indicating that hysteroscopic surgery, compared with no hysteroscopy prior to the first assisted reproductive technology (ART) cycle or following a failed attempt, significantly increased the live birth rate (RR 1.24, 95% CI 1.09–1.43, I^2^ = 21%) and the clinical pregnancy rate (RR 1.36, 95% CI 1.18–1.57; I^2^ = 51%) (32). The enhancement in clinical pregnancy rate was corroborated by subgroup analyses following implantation failure (RR 1.40, 95% CI 1.12–1.74, I^2^ = 52%) and prior to the first ART cycle (RR 1.32, 95% CI 1.11–1.57, I^2^ = 42%) (32).

However, no studies have specifically evaluated whether performing hysteroscopy before FET improves clinical outcomes in infertile patients with endometriosis. Hysteroscopic surgery is a widely utilized clinical procedure in assisted reproductive technology (33), primarily for diagnosing endometrial lesions and assessing uterine cavity conditions (34, 35). It is regarded as the gold standard for evaluating uterine cavity lesions and is effective in enhancing reproductive outcomes (36). By employing hysteroscopy to examine the uterine cavity, treat lesions, and address factors that impede embryo transfer, endometrial receptivity can be improved (14), potentially leading to better pregnancy outcomes following embryo transfer. Currently, no studies have explored whether hysteroscopic surgery prior to FET benefits infertile patients with endometriosis. This study represents the first single-center retrospective cohort analysis aimed at assessing the impact of hysteroscopic surgery prior to FET on pregnancy outcomes in patients with endometriosis. It aims to evaluate uterine cavity lesions in these patients and provide a reference for clinical diagnosis and treatment strategies in assisted reproductive technology for infertile patients with endometriosis.

## Materials and methods

### Research subject

This single-center retrospective cohort study focuses on endometriosis-related infertile patients who underwent their first *in vitro* fertilization-embryo transfer at the Reproductive Medicine Center of the Shanghai Changzheng Hospital between January 1, 2021, and May 31, 2025. The inclusion criteria were: (i) age between 23 and 40 years; (ii) body mass index (BMI) ≤ 28; and (iii) endometriosis was diagnosed via laparoscopy as the gold standard; when laparoscopy was not performed, the clinical diagnosis was made based on typical clinical features, including dysmenorrhea, ovarian endometrioma detected on ultrasonography (characterized by fine echogenic spots), and elevated serum CA-125 levels. The exclusion criteria were: (i) age >40 years; (ii) uterine malformations such as unicornuate or bicornuate uterus; (iii) immune system diseases requiring oral medication; (iv) severe adenomyosis or uterine fibroids distorting the uterine cavity contour (diagnosed via transvaginal ultrasound).

The study collected clinical data with the approval of the Medical Ethics Committee of the Shanghai Changzheng Hospital. Due to the retrospective nature of the research, the Ethics Committee waived the requirement for individual informed consent. (approval number: [2024SL138]; approval date: November 8, 2024. The approval letter specified a continuing review frequency of one year from the date of approval).

### Indications and contraindications of hysteroscopic surgery

Indications for hysteroscopic surgery were: (i) previous failed embryo transfer attempts at other hospitals; (ii) B-ultrasound showing hyperechoic masses or uneven endometrial echoes; (iii) Three-dimensional assessments revealing an irregular uterine cavity shape and disrupted endometrial line, which suggested intrauterine adhesions; (iv) ultrasound detection of uterine cavity effusion; and (v) fewer than 3 embryos, with the procedure depending on the patient's preference. Contraindications were: (i) acute inflammation of reproductive tract; and (ii) severe liver or kidney dysfunction, or systemic diseases that render the patient unsuitable for surgery.

### Procedure steps for hysteroscopic surgery

The operation was performed by two experienced surgeons from this reproductive medicine center. A 22Fr operative hysteroscope (Storz, Germany) was used, with normal saline as the uterine distension medium, and the uterine distension pressure was set at 120–130 mmHg. Following intravenous anesthesia, patients were positioned in the lithotomy position, and the cervix was gradually dilated to 6.5 mm using a cervical dilator. If cervical dilation was encountered to be difficult during the procedure, 40 mg of phloroglucinol was administered intravenously to soften the cervix before proceeding with hysteroscopic insertion. A hysteroscope was inserted to examine the uterine cavity comprehensively. Endometrial polyps were found and removed from the base of the polyps using 5Fr scissors or separation forceps. Hysteroscopic findings of micropolyps or strawberry-type endometrial congestion indicated potential chronic endometritis. Endometrial tissue was fully sampled using separation forceps. Uterine adhesions were found and adhesion release surgery was performed using 5Fr scissors.

Common complications of hysteroscopy included uterine perforation, fluid overload (or dilutional hyponatremia), and infection. To prevent uterine perforation, a preoperative bimanual pelvic examination was performed to ascertain the position and size, thereby guiding the standardized insertion of hysteroscopic instruments. In complex cases, such as severe intrauterine adhesions, intraoperative ultrasound monitoring was used to enhance procedural safety. To prevent fluid overload, we maintained the intrauterine distension pressure below 140 mmHg, limited fluid absorption to less than 1,000 mL, and completed the procedure within one hour. For infection prophylaxis, routine antibiotics were administered on the day of surgery and on the first postoperative day.

### Diagnosis and treatment of CE

CE required a comprehensive evaluation combining hysteroscopic examination and endometrial biopsy pathology. Suspicious hysteroscopic findings included endometrial micropolyps (≤1 mm in diameter) or the strawberry sign, characterized by diffuse endometrial hyperemia with scattered punctate white glandular openings on the surface. When hysteroscopic abnormalities were detected, endometrial tissue was obtained for CD138 immunohistochemical staining to specifically label stromal plasma cells. The pathological diagnostic criterion was the presence of ≥5 CD138-positive plasma cells in the endometrial stroma per 10 high-power fields (HPF) at ×400 magnification. Once diagnosed, patients received a 14-day course of oral doxycycline at a dosage of 100 mg twice daily (37).

### Frozen-thawed embryo transfer (FET)cycle

There were two endometrial preparations for the FET cycle: a hormone replacement therapy (HRT) and a down-regulation protocol utilizing GnRH agonists. In the down-regulation combined with hormone replacement cycle, a subcutaneous injection of 3.75 mg of gonadotropin-releasing hormone agonist (GnRH-a) (Beiyi, Shanghai Lihua) was administered on the second or third day of the menstrual cycle, followed by HRT after 28 days. In the HRT cycle, beginning on the second or third day of menstruation, oral doses of 2–6 mg/day of Femoston (estradiol/estradiol dydrogesterone combined tablets, 2 mg/10 mg, Netherlands) were prescribed. On the 14th to 16th day of hormone replacement, if the endometrial thickness exceeded 7 mm, endometrial transformation was initiated with a daily dosage of 90 mg of progesterone, along with 6 mg/day of Femoston yellow tablets for a duration of 4 to 6 days.

### Embryo cryopreservation, thawing and transfer

On the fifth day of endometrial transformation, cleavage-stage embryos were transferred, or blastocyst-stage embryos were transferred on the seventh day of transformation. Embryo thawing was carried out on the morning of the transfer day, and embryo transfer was performed 1 to 2 h after thawing. The number of embryos transferred was determined based on a comprehensive evaluation of patient age, embryo quality, cycle history, and patient preference, and was strictly in accordance with the *ESHRE guideline: number of embryos to transfer during IVF/ICSI* (38). All patients in this study received either 1 or 2 embryos per transfer. After embryo transfer, a daily dose of 90 mg of progesterone vaginal sustained-release gel (Crinone, Merck Serono, Switzerland) and 6 mg/day of Femoston yellow tablets were administered.

During embryo transfer, catheterization failure or embryo retention occasionally occurred. In cases of catheterization failure, the routine management involved exchanging the catheter for one with a rigid stylet or for a more rigid Wallace (H.G. Wallace Limited) transfer catheter. In cases of embryo retention, the retained embryo was confirmed by an embryologist under microscopy, then reloaded and retransferred.

### Pregnancy follow-up

Serum β-hCG testing was performed 14 days after embryo transfer. A β-hCG level of ≥5 U/L indicates biochemical pregnancy. On the 28th day after transfer, a vaginal ultrasound examination was conducted. The presence of an intrauterine gestational sac on transvaginal ultrasound confirmed clinical pregnancy. The conditions of all pregnant patients were followed up, including miscarriage, premature birth, live birth and multiple pregnancies.

### Observation indicators

The baseline clinical data, biochemical pregnancy rate, clinical pregnancy rate, miscarriage rate, live birth rate, preterm birth rate and multiple pregnancy rate were compared between groups. The main indicators were the clinical pregnancy rate and live birth rate, while the secondary indicators included biochemical pregnancy rate, miscarriage rate, preterm birth rate, and multiple pregnancy rate. The biochemical pregnancy rate is calculated as the number of biochemical pregnancy cycles divided by the total number of embryo transfer cycles, multiplied by 100%. The clinical pregnancy rate is calculated as the number of clinical pregnancy cycles divided by the total number of transfer cycles, multiplied by 100%. The calculation method for the miscarriage rate is the number of miscarriage cycles before 28 weeks of pregnancy divided by the number of clinical pregnancy cycles, multiplied by 100%. The calculation method for the live birth rate is the number of live births divided by the number of embryo transfer cycles, also multiplied by 100%. The calculation method for the preterm birth rate is the number of deliveries between 28 and 37 weeks of gestation divided by the number of live birth cycles multiplied by 100%. Lastly, the multiple pregnancy rate is the number of multiple pregnancy cycles divided by the number of clinical pregnancy cycles, multiplied by 100%.

### Statistical methods

Statistical analyses were performed with SPSS 27.0. Continuous variables with a normal distribution are presented as mean ± standard deviation (x ± s) and were compared using the independent-samples t-test. Continuous variables with a non-normal distribution are presented as median (25th percentile, 75th percentile) [M (Q1, Q3)] and were compared using the Mann–Whitney U rank-sum test. Categorical variables are reported as percentages (%) and were compared using the *χ*2 test; when the expected frequency T was less than 5, Fisher's exact test was applied. Propensity score matching (PSM) was conducted using R software version 4.3.3 (2024-02-29) to reduce baseline imbalance between the hysteroscopy and non-hysteroscopy groups. Propensity scores were estimated based on embryo attribute differences, which were considered the main imbalanced factor between groups. Patients were matched at a 1:1 ratio using a caliper width of 0.02. After matching, baseline characteristics and clinical outcomes were compared between the two groups using the same statistical methods described above. A two-sided *P* value < 0.05 was considered statistically significant.

## Results

### Basic characteristics of the two groups

In this study, 220 patients were analyzed, with 180 in the hysteroscopy group and 40 in the non-hysteroscopy group. We compared the groups based on age, BMI, number of deliveries, number of miscarriages, frozen embryo transfer cycle protocols, endometrial thickness on the day of luteal transformation, embryo attributes, and number of transferred embryos. Notably, significant differences were found in the embryo attributes between the groups. The hysteroscopy group exhibited a higher proportion of transferred blastocysts compared with the non-hysteroscopy group (*P* < 0.05). No statistically significant differences were observed in other baseline clinical data between the groups. Refer to Table 1 for detailed data.

**Table 1:** Baseline clinical data of the two groups of patients.

variable	Before PSM			After PSM		
	Hysteroscopy group (*n* = 180)	non-Hysteroscopy group (*n* = 40)	P	hysteroscopy group (*n* = 40)	non-hysteroscopy group (*n* = 40)	P
Age	33.00 (30.00, 36.00)	33.00 (30.00, 36.00)	0.843	34.00 (30.00, 35.25)	33.00 (30.00, 36.00)	0.839
BMI(Kg/m^2^)	21.00 (19.90, 22.90)	21.25 (19.75, 23.62)	0.692	21.45 (19.98, 24.02)	21.25 (19.75, 23.62)	0.747
Number of deliveries	0.00 (0.00, 1.00)	0.00 (0.00, 1.00)	0.406	0.00 (0.00, 1.00)	0.00 (0.00, 1.00)	0.947
Number of miscarriages	0.00 (0.00, 2.00)	0.00 (0.00, 1.00)	0.065	0.50 (0.00, 2.00)	0.00 (0.00, 1.00)	0.111
FET cycle protocols, (%)			0.336			0.606
GnRH-a + HRT	79.44 (143/180)	72.50 (29/40)		77.50 (31/40)	72.50 (29/40)	
HRT	20.56 (37/180)	27.50 (11/40)		22.50 (9/40)	27.50 (11/40)	
Endometrial thickness on the day of luteal transformation (mm)	10.00 (9.20, 11.47)	10.30 (9.05, 11.45)	0.944	9.70 (9.03, 11.20)	10.30 (9.05, 11.45)	0.683
Embryo attributes, (%)			0.013			1.000
Cleavage embryo	29.44 (53/180)	50.00 (20/40)		50.00 (20/40)	50.00 (20/40)	
Blastocyst embryo	70.56 (127/180)	50.00 (20/40)		50.00 (20/40)	50.00 (20/40)	
Number of transferred embryos, (%)			0.833			1.000
1	71.67 (129/180)	70.00 (28/40)		72.50 (29/40)	72.50 (29/40)	
2	28.33 (51/180)	30.00 (12/40)		27.50 (11/40)	27.50 (11/40)	

PSM was used to perform 1:1 matching for patients undergoing FET cycles, with embryo attribute differences as the core matching factor. A total of 80 patients were successfully matched and divided into the hysteroscopy group (*n* = 40) and the non-hysteroscopy group (*n* = 40). After matching, there were no statistically significant differences between the two groups, and the baseline data were well balanced. Refer to Table 1 for detailed data.

### Pregnancy outcomes

During the study's follow-up period, the hysteroscopy group demonstrated a higher biochemical pregnancy rate compared with the non-hysteroscopy group (68.89% vs. 52.50%, *P* < 0.05). Furthermore, the clinical pregnancy rate in the hysteroscopy group was significantly higher at 65.56%, in contrast to 42.50% in the non-hysteroscopy group (*P* < 0.05). Additionally, live birth rates were 55.56% for the hysteroscopy group and 35.00% for the non-hysteroscopy group, with this difference also reaching statistical significance (*P* < 0.05). However, no statistically significant differences were observed between the two groups regarding miscarriage rate (13.56% vs. 17.65%), preterm birth rates (7.00% vs. 21.43%), and multiple pregnancy rate (5.93% vs. 0.00%). For detailed data, refer to Table 2.

**Table 2:** Pregnancy outcomes of the two groups.

Variable	Before PSM			After PSM		
	hysteroscopy group (*n* = 180)	non-hysteroscopy group (*n* = 40)	P	hysteroscopy group (*n* = 40)	non-hysteroscopy group (*n* = 40)	P
Biochemical pregnancy rate	68.89 (124/180)	52.50 (21/40)	0.048	75.00 (30/40)	52.50 (21/40)	0.036
Clinical pregnancy rate	65.56 (118/180)	42.50 (17/40)	0.007	67.50 (27/40)	42.50 (17/40)	0.025
Live birth rate	55.56 (100/180)	35.00 (14/40)	0.019	55.00 (22/40)	35.00 (14/40)	0.072
Miscarriage rate	13.56 (16/118)	17.65 (3/17)	0.936	14.81 (4/27)	17.65 (3/17)	1.000
Preterm birth rate	7.00 (7/100)	21.43 (3/14)	0.199	13.64 (3/22)	21.43 (3/14)	0.658
Multiple pregnancy rate	5.93 (7/118)	0.00 (0/17)	0.595	11.11 (3/27)	0.00 (0/17)	0.418

The results of inter-group analysis after PSM matching showed that the biochemical pregnancy rate of patients in the hysteroscopy group was 75.00%, which was significantly higher than 52.50% in the non-hysteroscopy group, and the difference was statistically significant (*P* *<* 0.05). The clinical pregnancy rate of the hysteroscopy group was 67.50%, which was also significantly higher than 42.50% in the non-hysteroscopy group, with a statistically significant difference (*P* < 0.05). The live birth rate of the hysteroscopy group was 55.00%, which was higher than 35.00% of the non-hysteroscopy group, but this difference did not reach the level of statistical significance (*P* > 0.05). There were no statistically significant differences between the two groups in miscarriage rate, preterm birth rate and multiple pregnancy rate (all *P* > 0.05). For detailed data, refer to Table 2.

### Uterine cavity lesions in patients of the hysteroscopy group

The hysteroscopy results revealed that out of 180 patients before PSM, 31 (17.22%) exhibited no lesions, while 149 (82.78%) presented with lesions. Specifically, 39 patients (21.67%) were diagnosed with EP, 16 (8.89%) with CE, and 47 (26.11%) with intrauterine adhesions (IUA). Additionally, 23 patients (12.78%) had both IUA and EP, 13 (7.22%) had both IUA and CE, and 5 (2.78%) had both CE and EP. Notably, six patients (3.33%) were affected by CE, EP, and IUA simultaneously. For further details, please refer to Figure 1.

**Figure 1:**
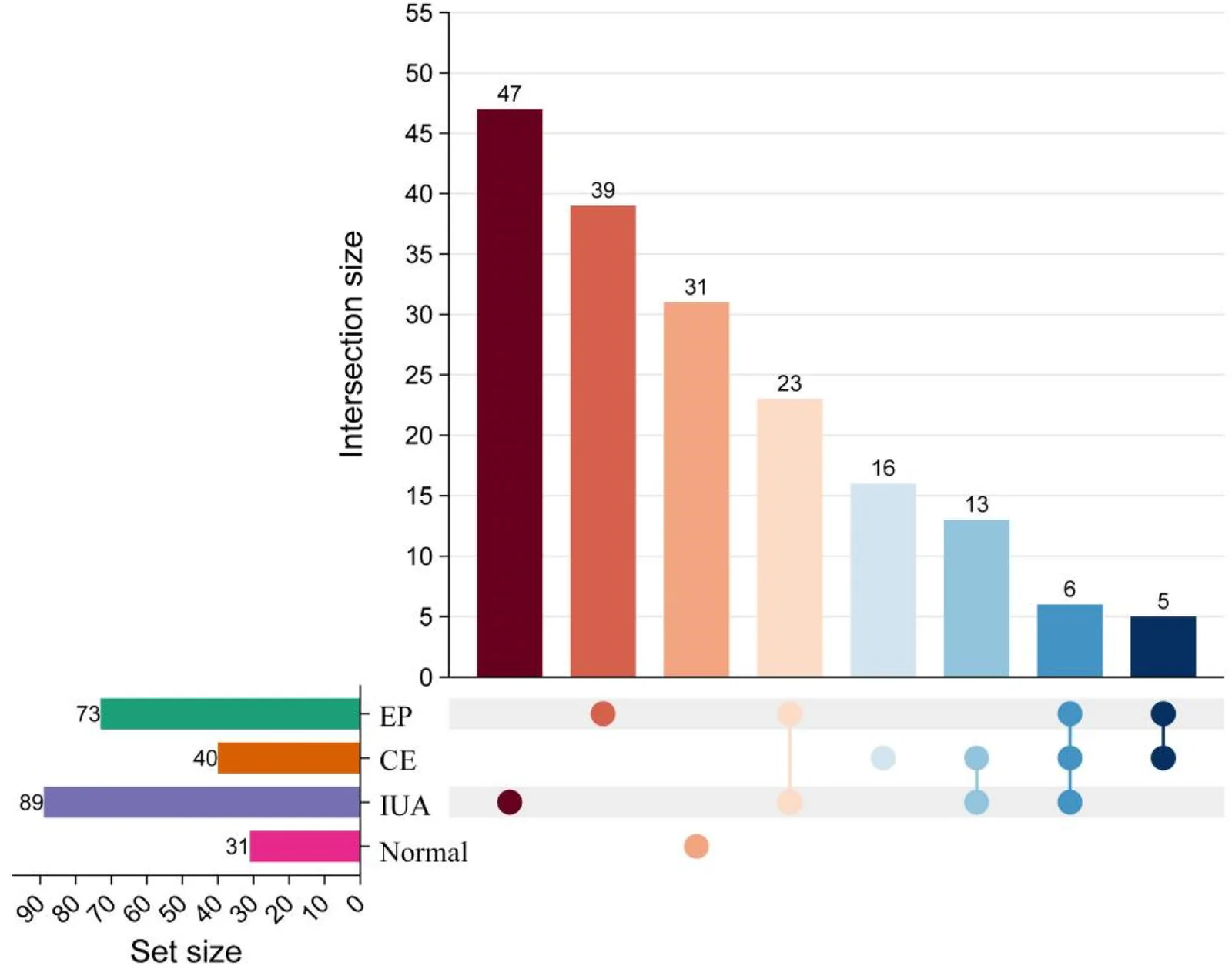
Upset diagram illustrating hysteroscopy examination outcomes before PSM.

After PSM, hysteroscopic findings showed that among the 40 patients, 5 (12.5%) had no detectable intrauterine lesions, whereas 35 (87.5%) were found to have lesions. Specifically, EP was identified in 12 patients (30%), CE in 6 patients (15%), and intrauterine adhesions (IUA) in 10 patients (25%). In addition, concurrent IUA and EP were observed in 4 patients (10%), while concurrent IUA and CE were found in 1 patient (2.5%). Notably, 2 patients (5%) presented with CE, EP, and IUA simultaneously. Further details are provided in Figure 2.

**Figure 2:**
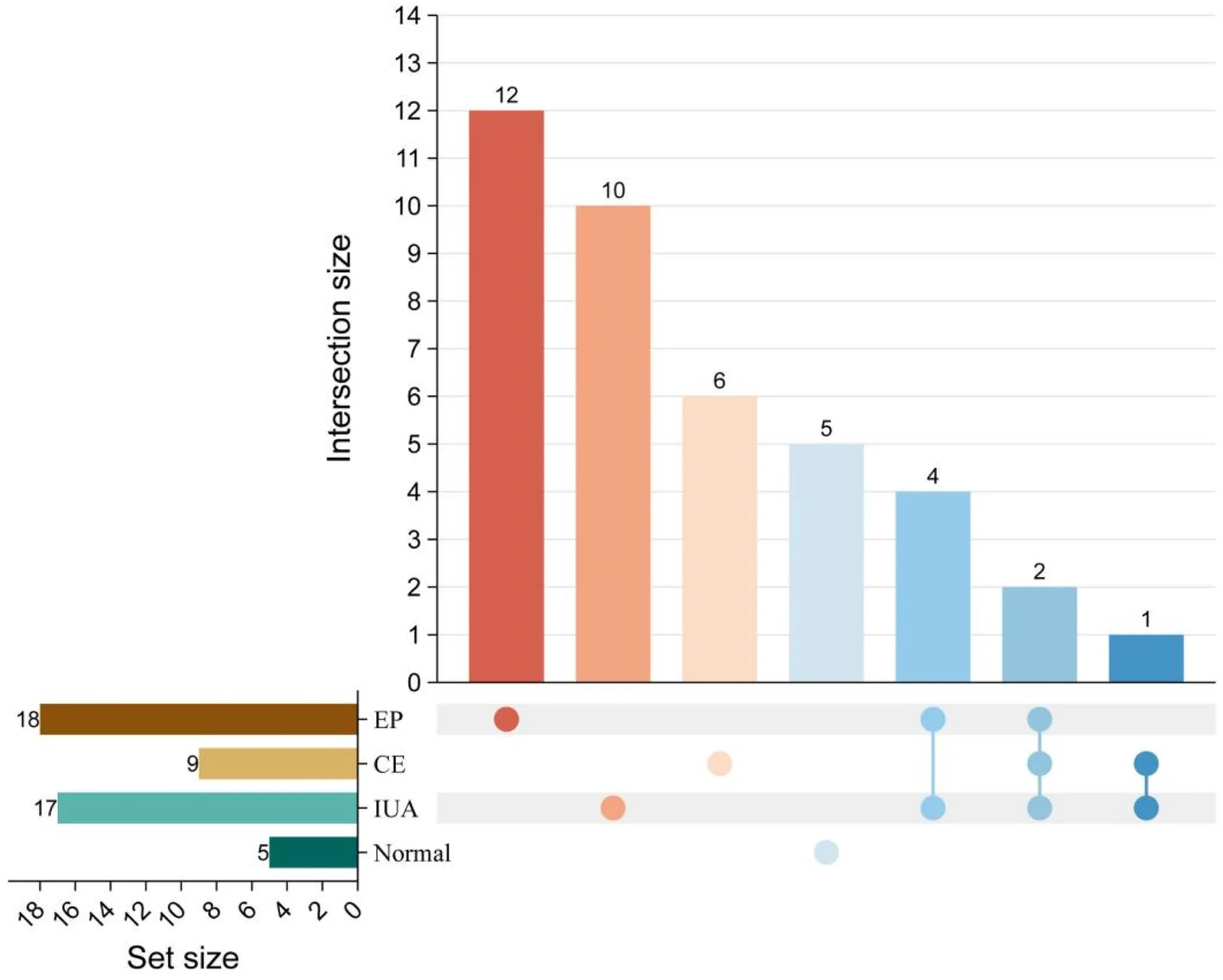
Upset diagram illustrating hysteroscopy examination outcomes after PSM.

## Discussion

Some studies have confirmed that hysteroscopy can improve the success rate of frozen-thawed embryo transfer in patients with uterine cavity lesions (33, 39). Our team's 2025 retrospective propensity-score matching cohort study, which included 879 patients undergoing their first FET, demonstrated that operative hysteroscopy significantly improved both clinical pregnancy rates and live birth rates in patients with suspected uterine cavity lesions (33). Specifically, after adjusting for confounding factors including age, BMI, delivery mode, number of miscarriages, fertilization method, FET protocol, embryo attributes, number of embryos transferred, and endometrial thickness, hysteroscopy was associated with a 54.3% increase in clinical pregnancy rates (OR 1.543, 95% CI 1.070–2.227) and a 44.2% increase in live birth rates (OR 1.442, 95% CI 1.011–2.056). These findings reinforced the clinical value of hysteroscopic evaluation and treatment in optimizing reproductive outcomes prior to embryo transfer. However, no studies have yet addressed whether hysteroscopy prior to FET improves clinical outcomes for infertile patients with endometriosis. This single-center retrospective cohort study represents the first investigation into the impact of hysteroscopy performed before the first FET cycle on pregnancy outcomes in this population.

The findings of this study demonstrated that the hysteroscopic group exhibited significantly higher rates of biochemical pregnancy and clinical pregnancy compared with the non-hysteroscopic group. Specifically, the hysteroscopy group had a significantly higher clinical pregnancy rate than the non-hysteroscopy group both before and after PSM [pre-PSM: 65.56% vs. 42.50%, *P* < 0.05; post-PSM: 67.50% vs. 42.50%, *P* < 0.05]. The live birth rate was likewise higher in the hysteroscopy group before and after PSM [pre-PSM: 55.56% vs. 35.00%, *P* < 0.05; post-PSM: 55.00% vs. 35.00%, *P* *>* 0.05], although the post-PSM difference did not reach statistical significance.

This study is the first to examine the effects of hysteroscopic surgery prior to the initial FET in infertile patients with endometriosis on pregnancy outcomes. While no other studies currently directly support our findings, existing research indicates that patients with endometriosis frequently exhibit a high likelihood of uterine cavity lesions, underscoring the importance of hysteroscopic surgery. Our study revealed that among 180 patients with endometriosis, 149 (82.78%) had lesions, with 73 (40.56%) presenting with EP and 40 (22.22%) with CE. Intrauterine adhesions were observed in 89 cases (49.44%). These findings align with earlier research. For instance, as early as 2003, Kim's study reported an EP incidence of 46.7% in patients with endometriosis, significantly higher than the 16.5% in the control group (40). Thus, it is strongly recommended that infertile patients with endometriosis undergo hysteroscopy to rule out EP, even if hysterosalpingography and transvaginal ultrasound do not indicate its presence. A meta-analysis found that the risk of EP among patients with endometriosis was 47.67%, compared with 14.97% in those without endometriosis (RR 2.81, 95% CI 2.48-3.18). EP risks for stage II-IV endometriosis were 61.46%, 61.64%, and 64.20%, respectively, which are slightly higher than the 51.38% reported for stage I (41). A potential link between the two conditions is plausible, since both are estrogen-dependent (42, 43). EP can cause infertility and failed embryo transfer through mechanisms like mechanical obstruction, local inflammatory responses in the endometrium, and decreased endometrial receptivity. CE is a mild but persistent inflammatory condition of the endometrium that has gained significant attention (24). It can alter the microenvironment and receptivity of the endometrium, impacting natural conception and potentially leading to complications such as assisted reproductive technology failure and miscarriage (16). Through re-staining endometrial pathology after hysterectomy, Takebayashi et al. reported that the prevalence of CE in patients with endometriosis was significantly higher compared with those without (52.94% vs. 27.02%) (20). Statistical analysis indicated that endometriosis is a strong predictor of CE, suggesting a robust connection between the two conditions. Qiao's retrospective study reported a 24.38% prevalence of CE among infertile patients with endometriosis (22). In comparison to women without CE, those diagnosed with CE exhibited notably lower cumulative pregnancy rates (46.51% vs. 71.13%) and live birth rates (44.19% vs. 63.38%) (22). Our study also identified 5 patients with EP, CE and intrauterine adhesions, along with 89 patients with intrauterine adhesions, all of which can contribute to embryo transfer failure. Consequently, we recommend hysteroscopic surgery and appropriate treatment for patients with endometriosis before proceeding with FET.

Although patients with endometriosis and infertility have a high likelihood of uterine cavity lesions, active hysteroscopic treatment can yield favorable pregnancy outcomes. These findings suggest that patients in the non-hysteroscopy group may possess unrecognized adverse factors, such as EP, CE, or intrauterine adhesions, which could hinder implantation and embryonic development. In contrast, hysteroscopic treatment of uterine cavity lesions prior to FET eliminates potential barriers to implantation, thereby enhancing clinical pregnancy.

This study offers three primary advantages. First, using a retrospective cohort design, it is the first to compare the effect of hysteroscopic surgery performed prior to the first FET on pregnancy outcomes in infertile patients with endometriosis, thereby clarifying the beneficial role of the surgery. Second, the study provides a comprehensive description of the hysteroscopic findings in these patients and documents a relatively high incidence of uterine cavity lesions, highlighting the necessity for hysteroscopic evaluation and treatment. Finally, all data were obtained from the Reproductive Medicine Center and the hysteroscopic procedures were performed by two experienced specialists, which enhances data reliability, ensures diagnostic accuracy, and minimizes selection bias.

Of course, this study has several limitations. First, owing to its retrospective design, the possibility of residual confounding and information bias cannot be completely excluded. Therefore, further prospective studies are needed to confirm the efficacy of hysteroscopic surgery in improving pregnancy outcomes following FET among infertile patients with endometriosis. Second, the sample size was relatively small, particularly in the non-hysteroscopy group, which may have limited the statistical power and generalizability of our findings. Although PSM was employed to balance observable baseline embryo variables and reduce confounding, residual selection bias derived from these unrecorded clinical factors cannot be excluded; therefore, our results should be interpreted with caution. Third, although early pregnancy assessments following FET were performed at our reproductive medicine center, subsequent antenatal, intrapartum, and postpartum care was not uniformly provided by our center for all patients. Because the quality and continuity of prenatal and perinatal care may influence pregnancy outcomes, particularly live birth and obstetric outcomes, their potential effects could not be fully controlled in the present retrospective study.

In conclusion, our study demonstrates a notable co-occurrence of endometriosis with EP and CE. Hysteroscopic management of these intrauterine lesions was associated with improved clinical pregnancy following FET in patients with endometriosis. Accordingly, evaluation for concurrent intrauterine lesions and appropriate hysteroscopic intervention should be considered when planning FET for patients with endometriosis.

## Data Availability

The raw data supporting the conclusions of this article will be made available by the authors, without undue reservation.
